# Screening and identification of human HPV18VLP neutralizing antibodies from women receiving HPV vaccine

**DOI:** 10.3389/fimmu.2025.1712231

**Published:** 2025-12-05

**Authors:** Yaru Gu, Rui Zhang, Wenhao Wang, Jinrui Zhou, Bixia Liu, Yan Zheng, Houyi Zuo, Yexiang Du, Ying Wang, Renjian Hu, Qianfei Zuo

**Affiliations:** 1 Department of Microbiology and Biochemical Pharmacy, College of Pharmacy, Army Medical University, Chongqing, China; 2 College of Pharmacy and Bioengineering, Chongqing University of Technology, Chongqing, China; 3 Department of Clinical Laboratory, General Hospital of Western Theater Command, People’s Liberation Army of China, Chengdu, China; 4 School of Pharmacy, Henan University, Kaifeng, China; 5 College of Medicine, Southwest Jiaotong University, Chengdu, China; 6 Department of Gastroenterology, Chongqing Key Laboratory of Digestive Malignancies, Daping Hospital, Army Medical University, Chongqing, China; 7 953th Hospital, Xinqiao Hospital, Army Medical University, Shigatse, China

**Keywords:** phage display technology, VLP18-Fab, cervical cancer, neutralizing antibody, molecular docking

## Abstract

Currently, the primary treatments for cervical cancer patients are surgery or chemoradiotherapy, with a lack of standardized pharmacological therapies. Here, we report the construction of a Fab fragment phage display antibody library with a capacity of 10^9, derived from peripheral blood mononuclear cells of 15 women vaccinated against HPV. Through four rounds of screening, one clone, VLP18-Fab, specifically binding to HPV18 virus-like particles (HPV18 VLP), was selected from 40 clones. Additionally, the VLP18-Fab antibody demonstrated a highly significant and dose-dependent neutralizing activity against HPV18 pseudoviruses (P < 0.0001).Moreover,following identification, the structure of the antibody-antigen binding complex was simulated using BIOVIA Discovery Studio, which included the identification of potential binding sites. Overall, this study underscores the potential of phage-displayed antibody library technology for screening neutralizing antibodies and provides valuable insights for the design of HPV vaccines based on structural studies of antigen-antibody complexes.

## Introduction

Cervical cancer ranks as the fourth deadliest cancer among women globally (1). According to GLOBOCAN data, in 2008, there were 529,000 new cases of cervical cancer worldwide, with 275,000 deaths (2). By 2020, the number of new cases increased to 604,000, with 342,000 deaths globally, placing cervical cancer at the top of the list of female cancer incidence or mortality in several countries (3). Both the incidence and mortality rates of cervical cancer are on the rise globally. Common treatment methods for cervical cancer include surgery, radiation therapy, and chemotherapy (4). Despite the availability of various treatments for cervical cancer, its disease burden remains a significant global issue, partly due to late diagnosis and limited access to care in some regions. Persistent infection with human papillomavirus (HPV) has been identified as a significant cause of cervical cancer and many other cancers (5). HPV screening and preventive vaccination programs have been initiated globally. However, although available prophylactic HPV vaccines effectively block HPV entry into cervical epithelial cells by generating HPV-specific neutralizing antibodies, their effectiveness may be limited in individuals already infected with certain HPV types (6). Additionally, the immunogenicity and protective efficacy of HPV vaccines can be suboptimal in individuals with an immunocompromised state. This includes those with congenital immunodeficiencies, HIV/AIDS, autoimmune diseases, or iatrogenic immunosuppression; for example, transplant recipients or patients undergoing chemotherapy. For these populations, a comprehensive clinical assessment focusing on immune status and overall health is recommended prior to vaccination to ensure safety and an adequate immune response.

Persistent HPV infection in the basal cells of the cervical epithelium is a primary risk factor for the development of cervical intraepithelial neoplasia or adenocarcinoma *in situ* (AIS). Without treatment, over several years to decades, a subset of women with these lesions progress from dysplasia to invasive cancer (7). Considering the repetitive cycle of HPV release from infected cells and subsequent infection of new cells in the cervical basal epithelium, neutralizing antibodies to prevent HPV infection may represent a feasible approach (8). Unlike vaccines, which typically take weeks to generate immune protection in vaccinated individuals, neutralizing monoclonal antibodies can offer immediate passive protection against viral infection (9). Therefore, neutralizing monoclonal antibodies are suitable for individuals of all ages. They are particularly beneficial for those at high risk who may not produce sufficient antibodies after vaccination, as well as for immunocompromised individuals. In developing countries, even with vaccines available, it may take years to achieve adequate immunization coverage in at-risk populations. Consequently, women in these regions will continue to be infected in the foreseeable future, highlighting the urgent need for effective therapeutic agents. Highly efficient and low-toxicity neutralizing monoclonal antibodies have been widely used to treat viral infections caused by respiratory syncytial virus, cytomegalovirus, human immunodeficiency virus, Ebola Virus, and influenza virus (10). The discovery and development of monoclonal antibodies that neutralize HPV may offer a promising approach to eliminate persistent HPV infection.

To date, more than 300 different types of human papillomavirus (HPV) have been identified (11), among which at least 12 are classified as “high-risk” strains, including HPV16, 18, 45, 33, 31, 58, 35, etc. which are the main culprits in cancer development (12). Among these high-risk HPV types, HPV18 is associated with the poorest cancer prognosis (13). Currently, effective neutralizing antibodies for the treatment of human papillomavirus type 18 (HPV18) have not been successfully developed. HPV is a non-enveloped, double-stranded DNA virus with an approximately 60-nm icosahedral capsid composed of the major L1 protein and the minor L2 protein. Notably, the major capsid protein of HPV, L1, can self-assemble into virus-like particles (VLPs). These VLPs mimic the shape and conformational epitopes of the viral capsid (14). To date, all analyzed HPV neutralizing monoclonal antibodies have been type-specific and target conformational epitopes within the highly variable loops exposed on the surface of HPV L1 VLPs (15). VLPs provide a platform for studying virus-like particle mimetics, which is crucial for the development of neutralizing antibodies and other therapeutic approaches. This research offers a robust foundation for improved prevention, diagnosis, and treatment of high-risk HPV infections and associated cancers.

Phage display technology is a molecular technique involving genetic modification of phage DNA that enables the expression of peptides, proteins, or antibody fragments on the surface of phages by fusing these molecules to phage coat proteins (16). This process involves the introduction of exogenous DNA sequences into specific positions of the phage genome. Upon phage infection, phage genes are expressed within the bacterial host. The inserted peptide or antibody fragments are displayed on the phage surface as fusion products with the encoded coat proteins (17).This process involves the introduction of exogenous DNA sequences into specific positions of the phage genome. Upon phage infection, phage genes are expressed within the bacterial host. The inserted peptide or antibody fragments are displayed on the phage surface as fusion products with the encoded coat proteins (18).Phage display technology has rapidly evolved due to its advantages, including short screening cycles, ease of operation, the acquisition of human-derived antibodies, and large-scale antibody production. Additionally, this technique enables the engineering of antibodies to achieve high affinity. As a result, as of 2020, nine human-derived monoclonal antibody drugs produced via phage display technology have been approved by the United States Food and Drug Administration (FDA) for the treatment of human diseases (19).

The aim of this study is to construct a phage antibody library against HPV18 virus-like particles (VLPs) using phage display technology. Through *in vitro* screening, we aim to obtain Fab antibodies with neutralizing activity against HPV18 VLPs, thereby providing an effective antibody-based therapeutic strategy for treating HPV18 infection. Additionally, we employed artificial intelligence methods to predict antigen-antibody structures and perform molecular docking to identify potential antigenic epitopes. These structural insights from antigen-antibody complexes offer valuable guidance for the design of subsequent HPV vaccines.

## Methods and materials

### Ethics statement

All procedures performed in this study adhered to the ethical standards of the Declaration of Helsinki. The study protocol received formal approval from the Ethics Committee of Army Medical University, and written informed consent was obtained from all volunteers enrolled in the study.

### Expression and purification of HPV18VLP

The pET-30a(+)-HPV18VLP plasmid was transformed into E. coli BL21(DE3) competent cells. A single colony was picked and inoculated into LB medium, then grown overnight at 37 °C before being diluted 1:1000 in fresh LB medium. The diluted culture was grown until the optical density at 600 nm (OD600) reached 0.6–0.8, and protein expression was induced by adding 0.2 mM IPTG at 16 °C for 16–20 h. Cells were harvested by centrifugation, resuspended, and lysed by high-pressure homogenization. The lysate was centrifuged and the supernatant was loaded onto a Ni-NTA affinity chromatography column. After incubation with the resin and washing to remove non-specifically bound proteins, the target protein was eluted using an imidazole gradient, concentrated by ultrafiltration, and stored at -80 °C.

### Identification of HPV18VLP positive sera

HPV18 VLP antigen (1 μg/ml) was used to coat ELISA plates overnight at 4 C. Plates were washed with phosphate-buffered saline containing 0.05% Tween 20 (PBST) and blocked with 3% BSA at 37 °C for 2h. Serum samples were added to the plates and incubated at 37 °C for 1h. After washing, goat anti-human IgG-HRP (1:10,000 dilution in PBST) was added and incubated for 45min. Following additional washes, TMB substrate was added and incubated for a specified time; then the reaction was stopped with sulfuric acid, and absorbance was measured at 450 nm.

### Construction of the Fab phage antibody library

Peripheral blood mononuclear cells (PBMCs) were isolated from HPV-vaccinated donors. Total RNA was extracted and reverse transcribed into cDNA. Variable (VH, Vλ, Vκ) and constant (CH1, Cλ, Cκ) region genes were amplified by PCR as described in (20). The fragments were assembled into full-length Fab (λ or κ) fragments via overlapping extension PCR. The Fab fragments and pComb3XTT vector were digested with SfiI, then ligated together, and the resulting constructs were electroporated into XL1-Blue competent cells. The transformed cells were rescued with helper phage VCSM13, and the Fab phage antibody library was precipitated using a PEG/NaCl solution.

### Library quality assessment

To assess the quality of the constructed Fab library, twenty individual colonies were randomly selected from the transformation plates. Plasmid DNA was extracted, and PCR amplification of the insert fragments was performed, followed by digestion of the PCR products with SfiI. The digestion products were analyzed by agarose gel electrophoresis to determine the insertion efficiency. Additionally, plasmid sequencing was performed to evaluate the library diversity based on sequence variation.

### Affinity selection (biopanning)

Four rounds of biopanning were performed to enrich for HPV18 VLP-specific binders. Immunotubes were coated overnight at 4 °C with HPV18 VLP antigen (50 µg/mL in 0.05 M carbonate buffer, pH 9.6) using decreasing coating volumes per round: 200 µL in round 1, 100 µL in round 2, 50 µL in round 3, and 30 µL in round 4. After blocking with 3% BSA-PBS, the phage display library was added and allowed to bind for 2 hours at 37 °C. Unbound phages were removed by washing ten times for 5 minutes each, with increasing stringency: PBS in round 1; 0.1% PBST in round 2; 0.2% PBST in round 3; and 0.3% PBST in round 4. Bound phages were eluted with Gly-HCl (pH 2.2), immediately neutralized, and used to infect log-phase E. coli XL1-Blue cells. The output phage titer was determined by plaque titration. The enriched phage pool was amplified for the next round of screening. Enrichment efficiency was monitored by calculating the ratio of input phage to output phage after each round.

### Analysis of enriched clones

Following the third and fourth rounds of panning, ten individual colonies were picked from the output plates. The inserts were analyzed by PCR, and restriction digestion according to the method described above. Positive clones were then sequenced to confirm diversity and identify unique sequences.

### Phage ELISA and soluble Fab expression

Clones from the third and fourth rounds of panning—a selection process used to enrich specific binders—were screened by phage display ELISA. Positive clones were sequenced, and soluble Fabs (VLP18-Fab) were expressed in E. coli HB2151 upon IPTG induction. Subsequently, periplasmic extracts were prepared for further analysis.

### Western blot and immunohistochemistry

Purified VLP18-Fab was analyzed by SDS-PAGE and Western blot using anti-His and anti-human Fab horseradish peroxidase (HRP) conjugated antibodies. For immunohistochemistry analysis, cervical cancer tissue sections, HPV18-positive, were deparaffinized and subjected to antigen retrieval. These sections were then incubated with VLP18-Fab, followed by detection with 3,3’-diaminobenzidine (DAB).

### Affinity measurement by biolayer interferometry

The binding affinity between VLP18-Fab and HPV18 VLP was quantitatively analyzed using an Octet RED96 system (Sartorius). Prior to analysis, both proteins were buffer-exchanged into PBS. His-tagged HPV18 VLP was immobilized on Anti-Penta-HIS biosensors using a 120-second loading step. The kinetic assay was performed as follows. First, biosensors were equilibrated in PBS for 60 seconds. Then, a 500-second association phase was conducted with serially diluted VLP18-Fab (ranging from 34 μM to 4.25 μM), followed by a 120-second dissociation phase in PBS. Data were analyzed using Octet Analysis Studio software version 12.0. Subsequently, sensorgrams were globally fitted to a 1:1 binding model to calculate the equilibrium dissociation constant (KD). The fitting quality was evaluated by χ² and R² values (both >0.95).

### Pseudovirus neutralization assay

Neutralization activity of VLP18-Fab was evaluated using an HPV pseudovirus-based assay. Briefly, 293FT cells were seeded in a 96-well plate at 4×10^4^ cells/mL, and preincubated for 2–6 hours under 5% CO_2_at 37 °C. HPV pseudovirus was diluted 1:1000 in complete DMEM. In a separate plate, VLP18-Fab was serially diluted in 3-fold steps (eight dilution steps) and mixed at 120 μL per well with 80 μL of diluted pseudovirus. Controls included virus control (VC: 80 μL medium + 80 μL pseudovirus), and cell control (CC: 160 μL medium). After 1 hour incubation at 4 °C, 100 μL of each mixture was transferred to the pre-seeded 293FT cells and incubated for 72 hours under 5% CO_2_ at 37 °C. Following incubation, neutralization was assessed by fluorescence microscopy.

### Structural modeling

The 3D structures of the VLP18-Fab complex and HPV18 VLP were predicted using AlphaFold2. Molecular docking was performed using Discovery Studio 2019 to model the interaction between the antigen and antibody and to identify potential epitopes.

### Statistical analysis

Unless otherwise specified, all experiments were performed with at least three independent biological replicates (n = 3). In neutralization assays, data normalization was carried out by scaling the signals relative to the virus control group (set as 100% infection) and the cell control group (set as 0% baseline signal). Comparisons of neutralization data between two groups were analyzed using a two-tailed unpaired Student’s t-test, while comparisons among multiple groups were performed by one-way ANOVA followed by Tukey’s honestly significant difference (HSD) *post-hoc* test. A p-value < 0.05 was considered statistically significant. All statistical analyses were conducted using GraphPad Prism software (version 8.0).

## Results

### Expression and purification of HPV18VLP

**Figure 1:**
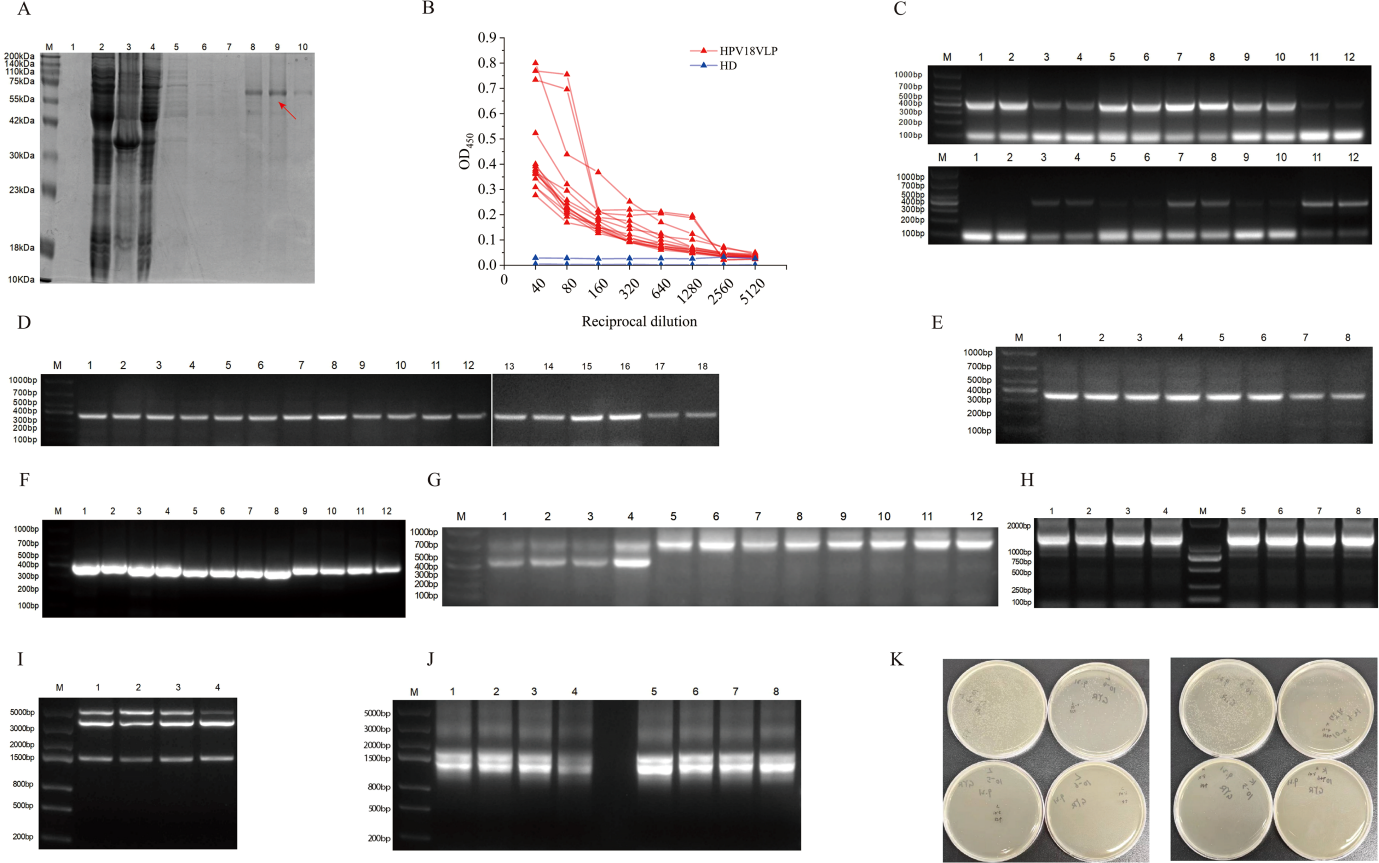
Construction of the VLP18-Fab phage antibody library. **(A)** SDS-PAGE analysis of HPV18 VLP protein purified by nickel-nitrilotriacetic acid (Ni-NTA) affinity chromatography. 1. Bacterial lysate supernatant before induction; 2. Bacterial lysate supernatant after induction; 3. Bacterial lysate pellet after induction; 4. Flow-through fraction; 5. High-salt wash fraction; 6–10. Elution fractions with 20 mM to 300 mM imidazole. **(B)** Serum analysis of 15 women vaccinated with HPV. **(C)** PCR amplification of 12 heavy chain variable regions (VH) genes. Adjacent wells were set up as duplicates. **(D)** PCR amplification of 9 light chain variable regions (Vλ) genes. Adjacent wells were set up as duplicates. **(E)** PCR amplification of 4 Vκ genes. Adjacent wells were set up as duplicates. **(F)** PCR amplification of human antibody constant regions: lanes 1–4 are heavy chain (CH1); lanes 5–8 are κ light chain (Cκ); lanes 9–12 are λ light chain (Cλ). **(G)** First-round overlap extension PCR: lanes 1–4 are VH-CH1; lanes 5–8 are Vκ-Cκ; lanes 9–12 are Vλ-Cλ. **(H)** Second-round overlap extension PCR: lanes 1–4 are Fabλ; lanes 5–8 are Fabκ. **(I)** Restriction digestion of pComb3XTT vector. **(J)** Restriction digestion of Fab fragments: lanes 1–4 are Fabλ; lanes 5–6 are Fabκ. **(K)** Determination of Fab phage antibody library size.

The Escherichia coli strain carrying the recombinant expression plasmid was induced with IPTG to express the HPV18 VLP antigen. After cell disruption by high-pressure homogenization, the supernatant was purified using Ni-NTA affinity chromatography. The purification results were then assessed by SDS-PAGE electrophoresis. A distinct band was observed at approximately 59 kDa on the protein gel after elution with 200 mM imidazole buffer, close to the expected molecular weight (**Figure 1A**). The SDS-PAGE gel was analyzed using Image Lab software to calculate the grayscale value of the target protein band, resulting in a purity of 85%. The protein concentration was determined to be 0.56 mg/mL using the BCA protein assay.

### Construction of the VLP18-Fab phage antibody library

Using HPV18 virus-like particles (HPV18 VLP) as the antigen, serum samples were collected from 15 women vaccinated against HPV for ELISA identification. Some samples showed elevated optical density (OD) values (**Figure 1B**). Peripheral blood mononuclear cells (PBMCs) were subsequently isolated from these 15 vaccinated women, and total RNA was extracted from the cells using Trizol reagent. Reverse transcription was then performed using a reverse transcription kit to obtain cDNA, which served as the template for PCR amplification of the human antibody heavy chain variable region genes. A total of 12 heavy chain variable region genes (**Figure 1C**), 9 λ light chain variable region genes (**Figure 1D**), and 4 κ light chain variable region genes (**Figure 1E**) were obtained, with relative molecular sizes ranging from approximately 300 to 400 bp. The constant region genes of the heavy and κ light chains were amplified using the pComb3XTT phage plasmid as a template. Meanwhile, the constant region gene of the λ light chain was amplified using the pComb3XLambda phage plasmid as a template. These amplifications resulted in fragments of approximately 300 to 400 bp in size (**Figure 1F**). Finally, the heavy chain variable region genes were fused with the heavy chain constant region genes, and the light chain variable region genes were fused with the light chain constant region genes through two rounds of overlap extension PCR. This process generated Fab gene fragments of approximately 1500 bp (**Figures 1G, H**).

The pComb3XTT vector and Fab gene fragments were separately subjected to Sfi I restriction endonuclease digestion and purified to obtain fragments with compatible sticky ends (**Figures 1I, J**). The vector and Fab gene fragments were then ligated using T4 DNA ligase to generate the recombinant vector pComb3XTT-Fab. Subsequently, the recombinant vector pComb3XTT-Fab was transformed into XL1-Blue competent cells via electroporation, resulting in the construction of the Fab phage antibody library. The Fabλ phage antibody library had a library size of 4.91×10^9, while the Fabκ phage antibody library had a library size of 2.92×10^9 (**Figure 1K**).

### Quality assessment of the Fab phage antibody library

**Figure 2:**
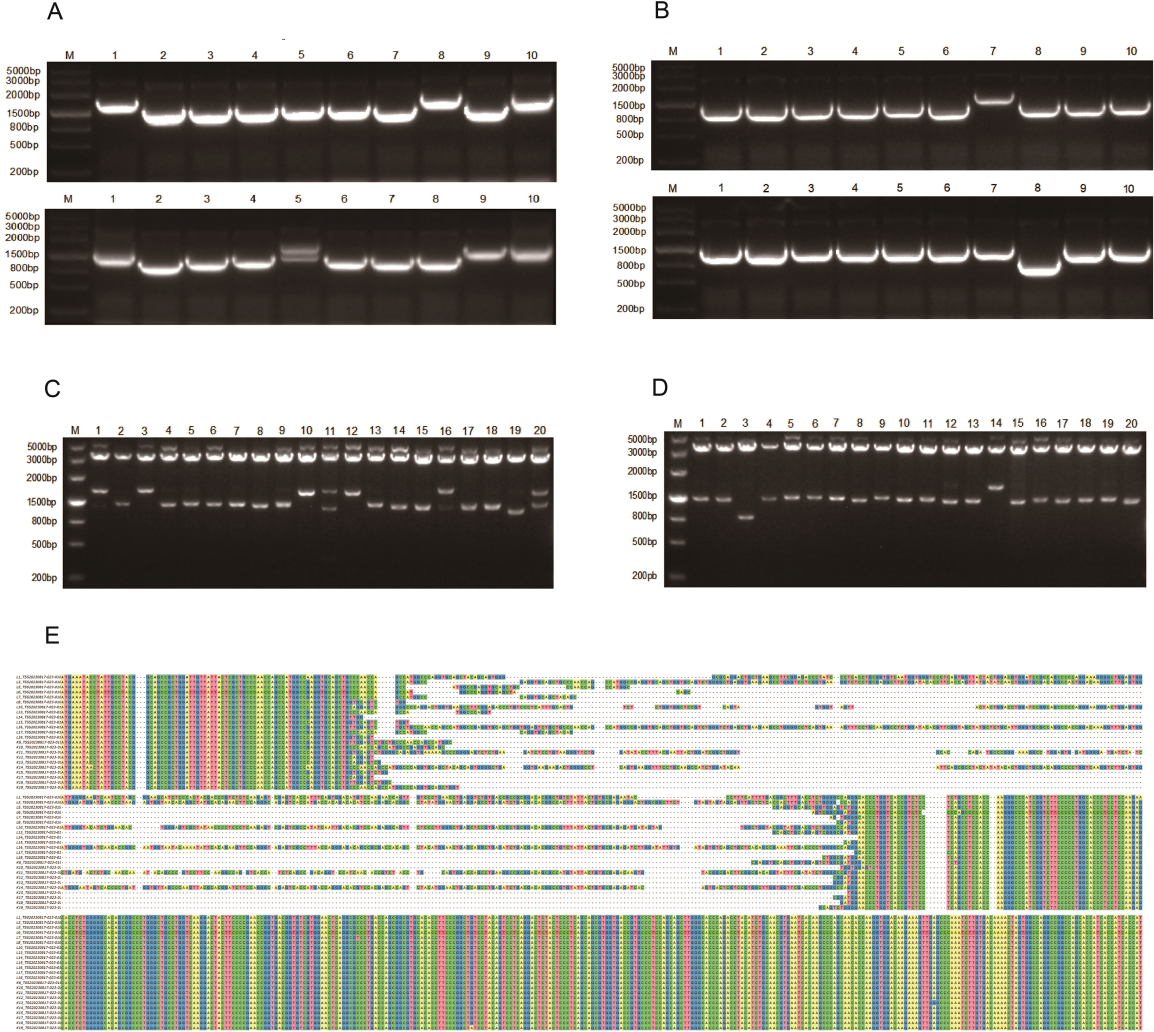
Quality assessment of the Fab phage antibody library. **(A)** PCR identification of 20 plasmids from the Fabλ phage antibody library; **(B)** PCR identification of 20 plasmids from the Fabκ phage antibody library; **(C)** Restriction enzyme digestion analysis of 20 plasmids from the Fabλ phage antibody library; **(D)** Restriction enzyme digestion analysis of 20 plasmids from the Fabκ phage antibody library; **(E)** Sequencing results showing sequence diversity of the Fab phage antibody library.

Twenty random colonies were selected from both Fabλ and Fabκ plates. Plasmids were then extracted for PCR identification to determine their PCR positive rates. The results showed targeted bands at approximately 1500 bp, with a PCR positive rate of 100% (**Figures 2A, B**). Subsequently, recombinant plasmids were digested with SfiI restriction endonuclease, and the digestion products were analyzed by 1% agarose gel electrophoresis. Two bands were consistently observed, corresponding closely to the sizes of the pComb3XTT vector fragment and the Fab gene fragment (**Figures 2C, D**), confirming the successful construction of pComb3XTT-Fab recombinant plasmids. Plasmids that tested positive in both PCR identification and digestion were subjected to sequencing analysis performed using Unipro UGENE and DNAMAN software. The homogeneity of the Fabλ phage antibody library was determined to be 66.84%, while that of the Fabκ phage antibody library was 56.69% (**Figure 2E**; complete sequences provided in **Supplementary Material S1**). Therefore, a diverse and high-quality Fab phage antibody library was successfully constructed.

### Four rounds of enrichment screening of the phage antibody library against HPV18VLP antigen

Four rounds of specific enrichment screening of the Fab phage antibody library were conducted using purified HPV18 VLP protein as the target antigen. In the first round, no additional screening pressure was applied, and only phosphate-buffered saline (PBS) was used for washing. Subsequently, in each round, the screening pressure was increased by reducing the antigen coating amount and increasing the Tween-20 concentration during the washing of unbound phages. This process of adsorption, washing, and amplification was repeated to select highly specific HPV18 VLP-Fab antibodies. By quantifying the input and output amounts through plate-based assays, the recovery rate was calculated to determine the number of screening rounds. The results showed that by the fourth round of screening, the recovery rate remained relatively stable, even showing a slight decrease. This led to the cessation of further screening. The phage antibody library was enriched by nearly a hundredfold (**Tables 1**, **2**).

**Table 1:** Enrichment effect of four rounds of selection on the Fabλ phage antibody library.

Number of selection rounds	Input (pfu)	Output (pfu)	Recovery rate
1 (PBS)	2×10^11^	3×10^5^	1.5×10^-6^
2 (0.1%PBST)	4.76×10^11^	3.8×10^6^	7.9×10^-6^
3 (0.2%PBST)	7×10^11^	4.2×10^7^	6×10^-5^
4 (0.3%PBST)	1.1×10^12^	4.19×10^7^	3.8×10^-5^

**Table 2:** Enrichment effect of four rounds of selection on the Fabκ phage antibody library.

Number of selection rounds	Input (pfu)	Output (pfu)	Recovery rate
1 (PBS)	4×10^11^	1×10^5^	2.5×10^-5^
2 (0.1%PBST)	2×10^11^	3.4×10^6^	1.7×10^-5^
3 (0.2%PBST)	9×10^11^	8.9×10^6^	9.8×10^-4^
4 (0.3%PBST)	1×10^12^	9.6×10^6^	9.6×10^-6^

### The results of the third and fourth rounds of screening of the phage antibody library were preliminarily identified

**Figure 3:**
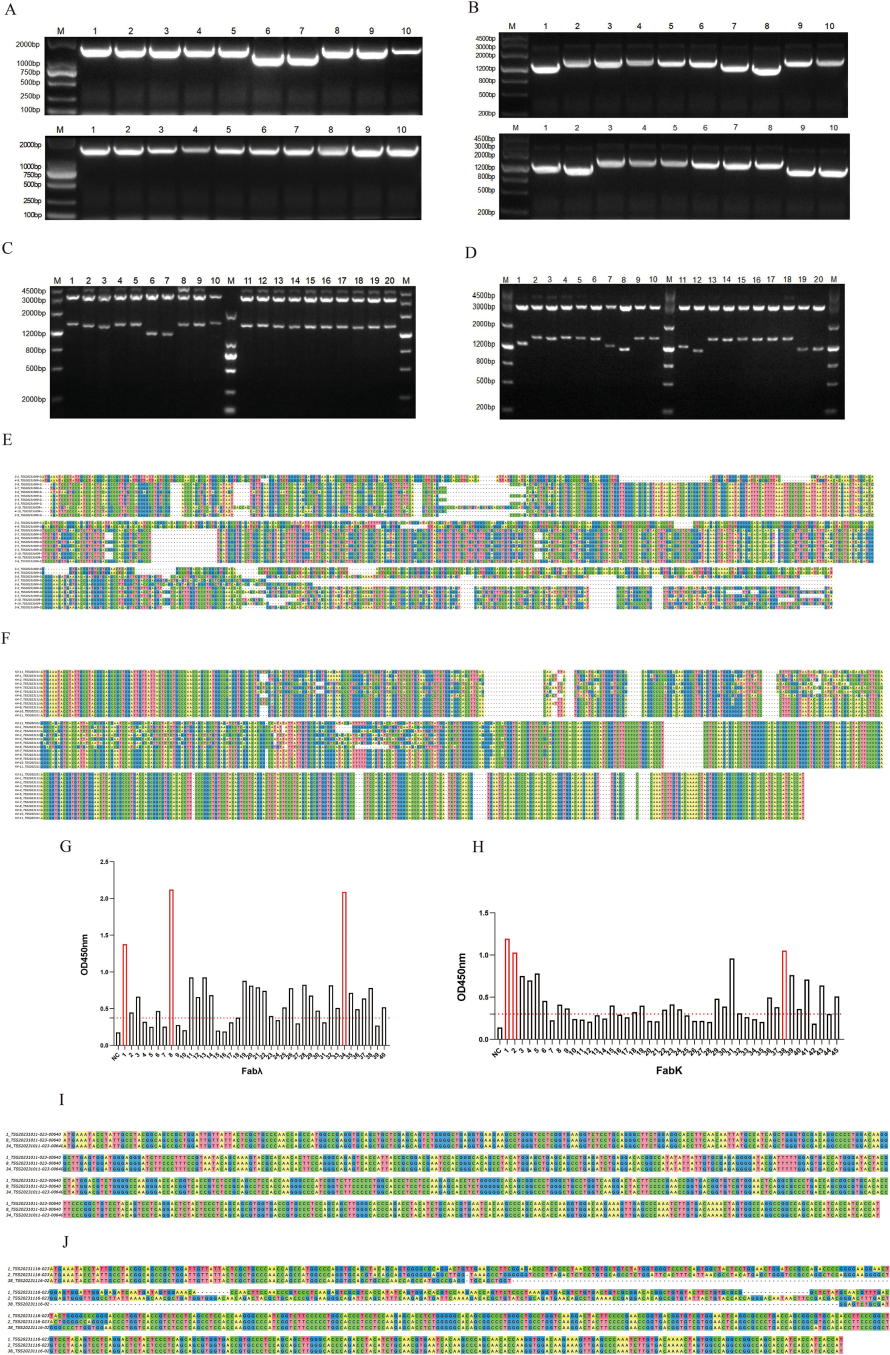
Preliminary characterization of phage antibody library screening results (rounds 3 and 4). **(A)** PCR analysis of Fabλ library clones. **(B)** PCR analysis of Fabκ library clones. **(C)** Restriction digestion analysis of Fabλ library clones. **(D)** Restriction digestion analysis of Fabκ library clones. **(E)** Sequencing results of Fabλ library clones. **(F)** Sequencing results of Fabκ library clones. **(G)** Phage-ELISA of Fabλ library clones. **(H)** Phage-ELISA of Fabκ library clones. **(I)** Sequencing results of the three positive clones with the highest OD values from the Fabλ phage antibody library. **(J)** Sequencing results of the three positive clones with the highest OD values from the Fabκ phage antibody library.

Ten single colonies were randomly selected from each of the third and fourth rounds of the Fabλ/κ phage antibody library plates for plasmid extraction. PCR and restriction enzyme digestion were performed separately, and all samples showed positive results as evidenced by 1% agarose gel electrophoresis (**Figures 3A–D**). Plasmids with positive results from both PCR and restriction enzyme digestion were subjected to sequencing analysis using the software Unipro UGENE and DNAMAN. The analysis revealed that the sequence homogeneity of the Fabλ phage antibody library increased to 80.82% after four rounds of screening, while that of the Fabκ phage antibody library increased to 82.22% (**Figures 3E, F**; complete sequences in S2). This further demonstrates the successful enrichment of the Fab phage antibody library.

### Phage-ELISA detection and sequencing of phage antibodies

After the third and fourth rounds of screening, 40 single colonies were randomly selected from the Fabλ plate and 45 single colonies from the Fabκ plate for inoculation and culture to prepare the phage antibody-containing supernatant. Phage-ELISA screening was performed with 3% BSA-PBS used as a negative control, and an OD450 value more than 2.1 times higher than the negative control was considered positive. The positive rates of Phage-ELISA screening for both the Fabλ and Fabκ phage antibody libraries were above 50% (**Figures 3G, H**). The top three clones with the highest absorbance values were selected for sequencing of Fab genes. The results showed that the Fab gene sequences of the three clones with the highest OD values in the Fabλ phage antibody library were identical. In contrast, the Fab gene sequences of the three clones with the highest OD values in the Fabκ phage antibody library were all different, with a homology of 84.84% (**Figures 3I, J**). Complete sequences are provided in **Supplementary Figure S3**.

### Expression and molecular weight determination of VLP18-Fab antibodies

**Figure 4:**
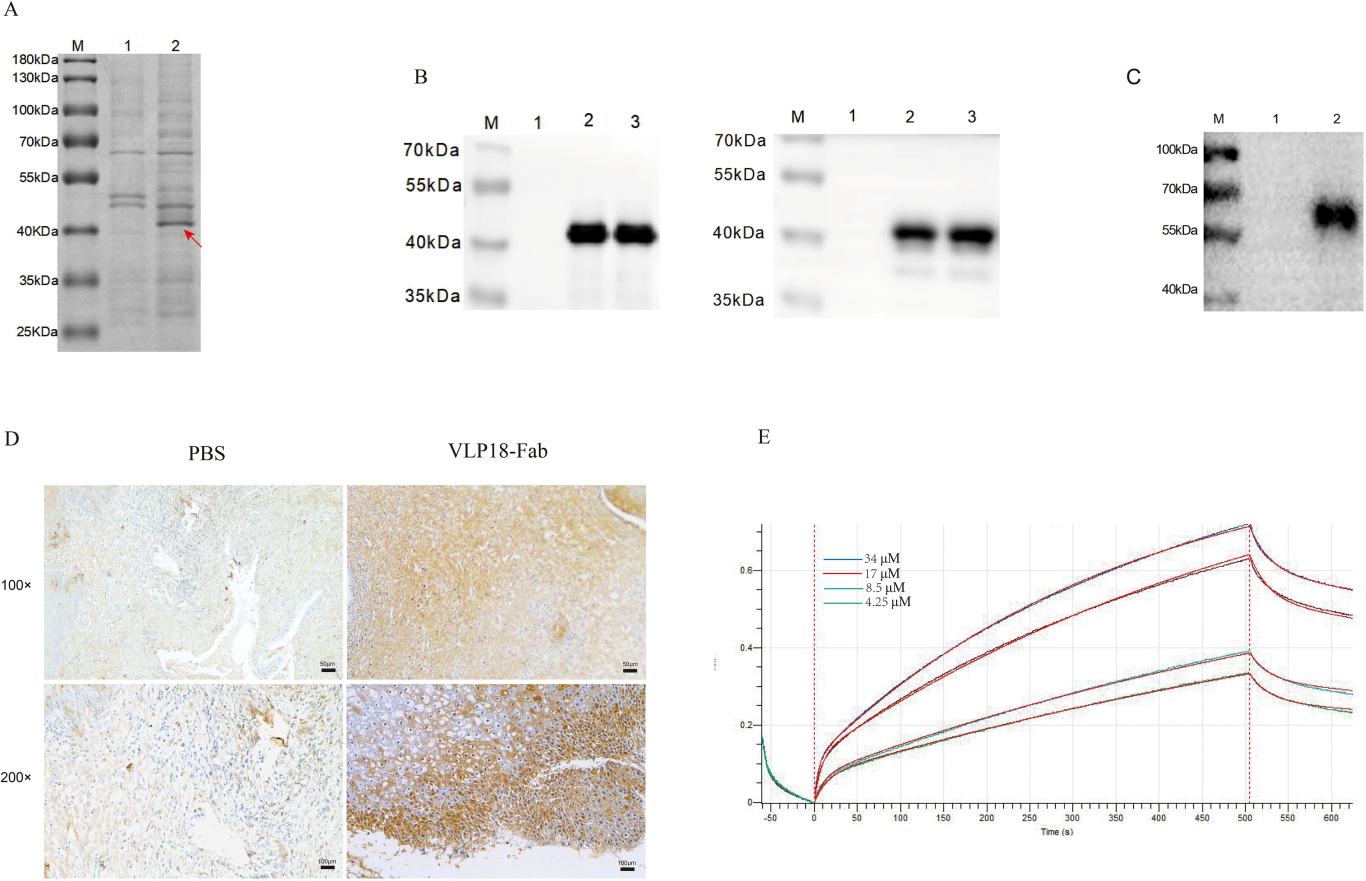
Soluble expression and characterization of VLP18-Fab antibodies. **(A)** SDS-PAGE analysis: lane M, molecular weight marker; lane 1, negative control (E. coli HB2151 empty vector); lane 2, VLP18-Fab expressed in **(E)** coli HB2151 (indicated by arrow). **(B)** Western blot probed with anti-His antibody (left) or anti-human Fab antibody (right): lane M, marker; lane 1, negative control; lanes 2–3, VLP18-Fab samples. **(C)** Western blot detection of antigen specificity: lane M, marker; lane 1, irrelevant antigen; lane 2, HPV18 VLP antigen. **(D)** Immunohistochemical analysis of cervical cancer tissue infected with HPV18 VLP: PBS-treated control group (left) and VLP18-Fab group (right). **(E)** Affinity measurement by bio-layer interferometry. Biotinylated HPV18 VLP was immobilized on streptavidin biosensors and assayed with multiple concentrations of VLP18-Fab. Equilibrium dissociation constants were derived from Octet system analysis.

Partial base deletions were observed in the gene sequences of the Fab segment from the three highest OD clones of the Fabκ phage antibody library, potentially resulting in frameshift mutations. Therefore, we expressed positive clones screened from the Fabλ phage antibody library to obtain functional Fab fragments. SDS-PAGE analysis revealed a distinct band at 42 kDa in the lysates of HB2151 cells infected with the positive clones, compared to the control strain HB2151 (**Figure 4A**). Since the pComb3XTT vector contains a 6×His-tag, we further confirmed the identity of the 42 kDa band as our target protein band. Western blot analysis of the expressed VLP18-Fab after SDS-PAGE and membrane transfer showed positive bands at 42 kDa when probed with both HRP-anti His-tag secondary antibody and rabbit anti-human IgG (Fab)-HRP secondary antibody (**Figure 4B**).

### Identification of the specificity and affinity of VLP18-Fab antibody towards HPV18 VLP

For Western Blot analysis, an irrelevant antigen was used as the negative control; VLP18-Fab served as the primary antibody, while rabbit anti-human IgG (Fab)-HRP served as the secondary antibody. The results demonstrated the specific binding capability of the VLP18-Fab antibody towards HPV18 VLP protein (**Figure 4C**). To further validate the specificity of VLP18-Fab towards HPV18 VLP, we employed quantitative fluorescence PCR to detect HPV18 VLP in paraffin-embedded cervical cancer patients’ tissues. Compared to the PBS group, the detection results with VLP18-Fab showed positive signals (**Figure 4D**). The affinity of the VLP18-Fab antibody towards HPV18 VLP was confirmed using Bio-Layer Interferometry (BLI). Upon biotin labeling of HPV18 VLP, the affinity between the VLP18-Fab antibody and HPV18 VLP was determined to be within the micromolar range, with a KD value of 1.002 × 10^−5^ M (**Figure 4E**). Taken together, these findings elucidate the excellent specificity and affinity of the VLP18-Fab antibody towards HPV18 VLP.

### Neutralization activity assay of VLP18-Fab antibody

**Figure 5:**
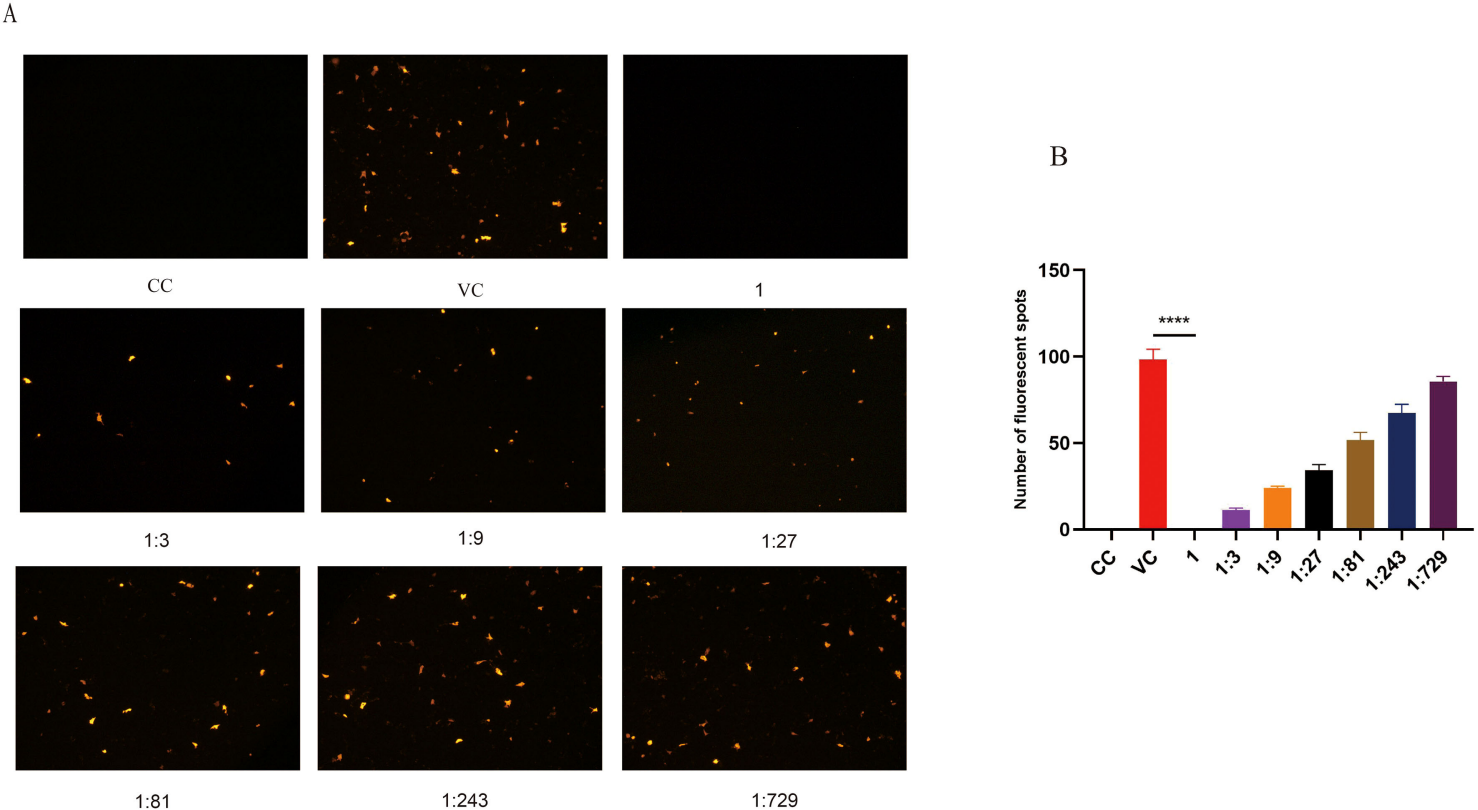
Evaluation of the neutralizing activity of the VLP18-Fab antibody. **(A)** Schematic of the neutralization assay. Serially diluted VLP18-Fab antibodies were incubated with the virus. The reduction in infection was assessed by counting fluorescent foci. CC, cell control; VC, virus control. **(B)** Quantitative analysis of fluorescent foci. The difference in fluorescent foci count between the virus control group and the antibody-treated groups was statistically significant (P < 0.0001). **** indicates a statistically significant difference (p < 0.0001) in neutralizing activity between the VLP18-Fab antibody group and the virus control group.

The neutralizing activity of the VLP18-Fab antibody was assessed using human papillomavirus pseudoviruses. After preparing serial dilutions of the VLP18-Fab antibody at ratios of 1:1, 1:3, 1:9, 1:27, 1:81, 1:243, and 1:729, the antibody was incubated with HPV18 pseudoviruses before being added to 293FT cells. The VLP18-Fab antibody demonstrated potent and dose-dependent neutralization activity against HPV18 pseudoviruses, with a high degree of statistical significance (P < 0.0001), as shown in **Figures 5A, B**, providing preliminary evidence for its specific neutralizing function.

### Prediction and molecular docking of VLP18-Fab and HPV18VLP three-dimensional structures

**Figure 6:**
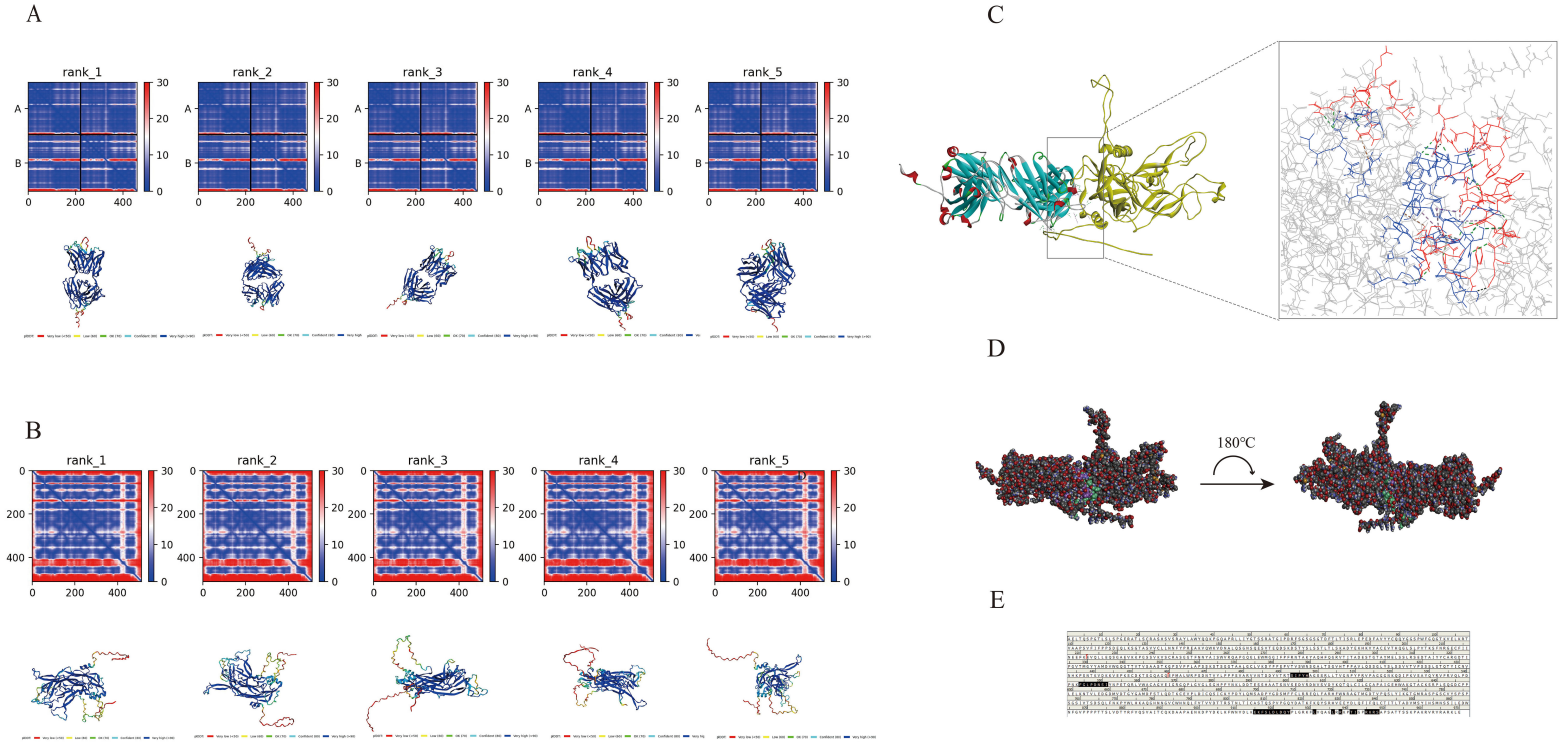
Structure prediction and molecular docking of VLP18-Fab with HPV18 VLP. **(A)** Predicted structural models of VLP18-Fab, ranked by pLDDT scores (Ranks 1–5). **(B)** Predicted structural models of HPV18 VLP, ranked by pLDDT scores (Ranks 1–5). **(C)** Three-dimensional model of the molecular docking between antibody VLP18-Fab (colored in blue, red, and green) and antigen HPV18VLP (shown in yellow). **(D)** Atomic-resolution structural model of the molecular docking between antibody VLP18-Fab and antigen HPV18 VLP, with the binding site on the antibody colored purple and the corresponding site on the antigen colored green. **(E)** Predicted epitopes on HPV18 VLP: black labels indicate key interacting residues.

The three-dimensional structures of VLP18-Fab antibodies and HPV18VLP were predicted using the AlphaFold2 program. The predicted structures of VLP18-Fab antibodies, ranked from 1 to 5 based on pLDDT scores, along with their corresponding three-dimensional models, were shown in (**Figure 6A**). Similarly, the predicted structures of the antigen HPV18VLP, ranked from 1 to 5 based on pLDDT scores, along with their corresponding three-dimensional models, were presented in (**Figure 6B**). We used the Discovery Studio 2019 program to construct the three-dimensional complex structure of the VLP18-Fab fragment and HPV18VLP antigen. The potential epitopes on HPV18VLP recognized by the VLP18-Fab fragment were predicted and are shown in (**Figures 6C, D**). The epitopes of HPV18VLP included Ser^493^, Ile^494^, Phe^495^, Tyr^496^, His^497^, Phe^544^, Gly^545^, Leu^546^, Pro^547^, Asp^548^, Asn^549^, Ser^550^, Ile^551^, Glu^915^, Lys^916^, Phe^917^, Ser^918^, Leu^919^, Asp^920^, Leu^921^, Asp^922^, Gln^923^, Asp^924^, Leu^931^, Leu^936^, Arg^938^, Thr^941^, Ile^942^, Arg^945^, Lys^946^, Arg^947^, and Ser^948^, which are involved in the interaction between VLP18-Fab and the antigen (**Figure 6E**).

## Discussion

Phage display technology stands as a pivotal platform for the development of human antibodies. It has been successfully employed to present diverse antibody formats such as single-chain variable fragments (scFvs), antigen-binding fragments (Fabs), and variable domains of heavy-chain-only antibodies (VHH). While scFvs benefit from low immunogenicity, small molecular weight, and rapid *in vivo* clearance, their clinical translation is often hampered by issues of inadequate stability, reduced affinity due to the lack of an Fc region, and a short serum half-life. In contrast, Fabs demonstrate superior structural stability and antigen-binding capacity, more closely mimicking the binding characteristics of intact IgG antibodies (21).They exhibit enhanced stability in phage display systems (22) and a lower propensity for *in vivo* oligomerization (23),which confers favorable pharmacokinetic properties and tissue penetration.

Given these advantages, our study focused on constructing a human Fab phage antibody library; this approach aims to overcome the stability and affinity limitations of scFvs and to provide a robust foundation for discovering therapeutic antibodies.

Despite the profound success of preventive HPV vaccines, global coverage remains incomplete due to factors such as cost, accessibility, and variable immune responses. The significant global burden of cervical cancer—with approximately 604,000 new cases and 342,000 deaths in 2020—highlights the urgent need for effective therapeutic agents. Since persistent HPV infection is the primary cause of cervical neoplasia, neutralizing antibodies offer a promising strategy to block viral entry into epithelial cells. This, in turn, prevents malignant transformation. Although neutralizing antibodies have been successfully deployed against other viruses, such as RSV and Ebola, the development of potent HPV-neutralizing antibodies is still an emerging field. In this context, we constructed an HPV18 virus-like particle (VLP)-specific fragment antigen-binding (Fab) phage display library and successfully isolated VLP18-Fab, a Fab fragment demonstrating neutralizing activity *in vitro*, as confirmed by cell infection inhibition assays. This represents a significant step toward a potential antibody-based therapy for HPV18 infections. Future work could involve cloning selected Fab genes into eukaryotic expression vectors to overcome the limitations associated with prokaryotic systems (24, 25).

Our preliminary molecular docking analysis offers a predictive and exploratory model of the VLP18-Fab and HPV18 VLP interaction. The generated epitope residue list provides a testable hypothesis and a blueprint for future mechanistic studies focused on antibody-antigen interactions. To build upon these findings, we propose a comprehensive roadmap for future work. First, to precisely quantify the neutralizing potency of VLP18-Fab, we will determine its exact IC50 value through complete dose-response curves. This approach will enable direct comparison with other reported HPV18 neutralizing antibodies. Subsequent in-depth characterization will necessarily incorporate isotype controls and previously reported positive neutralizing antibodies as references, to enable rigorous parallel comparisons. Second, to experimentally validate the predicted binding interface, we will employ site-directed mutagenesis (e.g., alanine scanning) of key residues, followed by affinity assessment using surface plasmon resonance (SPR). The ultimate goal will be to resolve the high-resolution structure of the complex using cryo-electron microscopy, which will definitively reveal the precise neutralization mechanism.

While the current library derived from 15 donors proved sufficient for isolating a functional antibody, we acknowledge that a larger donor cohort (n > 50) would further enhance library diversity, potentially yielding antibodies with higher affinity or broader cross-reactivity against HPV variants. With continuous advancements in high-capacity library technology—characterized by increased library size and complexity—improved transformation efficiency, and optimized screening strategies, antibody library platforms are maturing rapidly. This progress makes the isolation of high-quality antibodies against specific antigens increasingly feasible. Notably, several fully human antibody drugs developed via phage display technology have been successfully marketed, offering new therapeutic options for various diseases. Although an initial Fabκ candidate from our library was not successfully expressed due to a frameshift mutation, this setback highlights areas for improvement. Therefore, future efforts will focus on methodological refinements, including primer redesign, increased library diversity, enhanced electroporation efficiency, and improved antigen purity to bolster library quality. Antibody libraries are designed to mimic the *in vivo* affinity maturation process; however, because this process is simulated *in vitro*, the initial antibody isolates often exhibit only moderate affinity. Therefore, alongside library optimization, antibody engineering techniques such as chain replacement (26) and site-directed mutagenesis (27)are essential for affinity enhancement. By increasing expression yield, improving affinity, and conducting thorough *in vivo* validation, we anticipate that these antibodies can be developed into clinically viable therapeutics. These efforts will be complemented by advanced computational methods, such as molecular dynamics simulations and binding free energy calculations, which will precisely map energy contributions and identify key hotspot residues for optimization. Finally, the therapeutic efficacy of the optimized VLP18-Fab will be evaluated in an HPV18-induced disease model, such as K14-HPV18 transgenic mice. We will test local administration routes, for example, vaginal gel, to closely mimic potential clinical applications in high-risk patients.

In conclusion, this study establishes a foundation for developing HPV18-neutralizing antibodies using a Fab phage display platform. The integrated approach—comprising *in vitro* screening, computational prediction, and future directions focused on mechanistic validation and affinity maturation—provides a solid framework for advancing VLP18-Fab through preclinical development toward a potential clinical therapeutic. This work also contributes to the rational design of future HPV vaccines.

## Data Availability

The original contributions presented in the study are included in the article/**Supplementary Material**. Further inquiries can be directed to the corresponding authors.
