# Supplementary material for: Screening and identification of human HPV18VLP neutralizing antibodies from women receiving HPV vaccine
Source: Front Immunol. 2025 Dec 5;16:1712231. doi: 10.3389/fimmu.2025.1712231 (PMC12714908; doi:10.3389/fimmu.2025.1712231)
Supplement: Supplementary file 1 [file DataSheet1.docx]

***Supplementary Material***

A

B

Fig.S1. The complete sequences of the constructed Fab phage antibody libraries were obtained through sequencing. (A) The homogeneity of the Fabλ phage antibody library was 66.84%. (B) The homogeneity of the Fabκ phage antibody library was 56.69%.

A

B

Fig.S2. Complete sequencing of the third and fourth rounds of screening in the phage antibody library. (A) After four rounds of screening, the homogeneity of the Fabλ phage antibody library increased to 80.82%. (B) After four rounds of screening, the homogeneity of the Fabκ phage antibody library increased to 82.22%.

A

B

Fig. S3. Complete sequences of the three highest OD value positive monoclonal from Phage-ELISA results. (A) The nucleotide sequences of the Fab segment genes of the three highest OD value positive monoclonal in the Fabλ phage antibody library are identical. (B) The nucleotide sequences of the Fab segment genes of the three highest OD value positive monoclonal in the Fabκ phage antibody library are different, with a homogeneity of 84.84%.
